# Detection of Low-Abundance KRAS Mutations in Colorectal Cancer Using Microfluidic Capillary Electrophoresis-Based Restriction Fragment Length Polymorphism Method with Optimized Assay Conditions

**DOI:** 10.1371/journal.pone.0054510

**Published:** 2013-01-23

**Authors:** Huidan Zhang, Jin Song, Hui Ren, Zhangrun Xu, Xiaonan Wang, Lianfeng Shan, Jin Fang

**Affiliations:** 1 Department of Cell Biology, Key Laboratory of Cell Biology, Ministry of Public Health, and Key Laboratory of Medical Cell Biology, Ministry of Education, China Medical University, Shenyang, China; 2 Department of Colon Surgery, Shenyang Anal Rectum Hospital, Shenyang, China; 3 Research Center for Analytical Sciences, Northeastern University, Shenyang, China; 4 Department of Mathematics, College of Basic Medical Sciences, China Medical University, Shenyang, China; The Chinese University of Hong Kong, Hong Kong

## Abstract

Constitutively active KRAS mutations have been found to be involved in various processes of cancer development, and render tumor cells resistant to EGFR-targeted therapies. Mutation detection methods with higher sensitivity will increase the possibility of choosing the correct individual therapy. Here, we established a highly sensitive and efficient microfluidic capillary electrophoresis-based restriction fragment length polymorphism (µCE-based RFLP) platform for low-abundance KRAS genotyping with the combination of µCE and RFLP techniques. By using our self-built sensitive laser induced fluorescence (LIF) detector and a new DNA intercalating dye YOYO-1, the separation conditions of µCE for ΦX174 HaeIII DNA marker were first optimized. Then, a Mav I digested 107-bp KRAS gene fragment was directly introduced into the microfluidic device and analyzed by µCE, in which field amplified sample stacking (FASS) technique was employed to obtain the enrichment of the RFLP digestion products and extremely improved the sensitivity. The accurate analysis of KRAS statuses in HT29, LS174T, CCL187, SW480, Clone A, and CX-1 colorectal cancer (CRC) cell lines by µCE-based RFLP were achieved in 5 min with picoliter-scale sample consumption, and as low as 0.01% of mutant KRAS could be identified from a large excess of wild-type genomic DNA (gDNA). In 98 paraffin-embedded CRC tissues, KRAS codon 12 mutations were discovered in 28 (28.6%), significantly higher than that obtained by direct sequencing (13, 13.3%). Clone sequencing confirmed these results and showed this system could detect at least 0.4% of the mutant KRAS in CRC tissue slides. Compared with direct sequencing, the new finding of the µCE-based RFLP platform was that KRAS mutations in codon 12 were correlated with the patient’s age. In conclusion, we established a sensitive, fast, and cost-effective screening method for KRAS mutations, and successfully detected low-abundance KRAS mutations in clinical samples, which will allow provision of more precise individualized cancer therapy.

## Introduction

Colorectal cancer (CRC) represents a major public health problem due to its high incidence and mortality rate. Especially, the metastatic CRC (mCRC) is the leading cause of cancer-related deaths [1]. Over the last decade, treatment for mCRC has evolved from single agent 5-fluorouracil (5FU) to combination chemotherapy, and more recently to the inclusion of monoclonal antibodies (mAbs) such as cetuximab and panitumumab which can block the extracellular domain of the epidermal growth factor receptor (EGFR) and has significantly improved the median overall survival to 2 years and the 5-year survival to 10% [1]–[5]. However, patients with CRC who express a mutated version of the KRAS gene won’t benefit from this expensive targeted therapy and might be exposed to some side effects [1]–[6]. The KRAS proto-oncogene encodes a P21 protein which is a key downstream effector of EGFR and plays a critical role in controlling signal transduction pathways during cell growth. Activating mutations of KRAS is the most common oncogenic alteration in various human cancers. In CRC, constitutively active KRAS mutation has been found to render tumor cells independent of EGFR signaling and thereby resistant to EGFR-targeted therapies [7]–[10].

In 2009, the U.S. Food and Drug Administration (FDA) and European Medicines Agency approved labeling changes to cetuximab and panitumumab indicating that these agents are not recommended for the treatment of mCRC harboring KRAS mutations. Therefore, *KRAS* gene status becomes a *mandatory* prerequisite to mCRC therapy, and accurate detection of KRAS mutations in these patients is an urgent need. DNA sequencing is considered the gold standard for KRAS mutation detection, but the high cost and laborious operations prevent it from being employed universally [11]. Moreover, considering cancer-derived samples often consist of highly heterogeneous mixtures of stromal cells and cancer cells, lower sensitivity of sequencing analysis (20%) tends to cause false negative results and misconduct the clinical therapy [12]. Consequently, a variety of more sensitive PCR-based screening techniques have been developed, and indicated that methods with high sensitivities could achieve higher mutation detection rate, which will in turn improve the CRC treatment [13]–[15]. Because only limited hotspot mutations in KRAS codons 12 and 13 are proved to be related to the clinical medicine [16], the simple and cost-effective PCR/restriction fragment length polymorphism (RFLP) analysis has been extensively used to detect KRAS mutations [17]–[20], which allows the reliable discrimination between wild-type sequences and homozygous or heterozygous point *mutations* by generating or destructing restriction sites through PCR and subsequent electrophoresis [21]. At present, several commercial kits based on this principle are available and have been applied to reveal mutant KRAS, but their sensitivities just equal to or a little higher than sequencing [22], [23]. Some modified methods have also been developed to increase the sensitivity, such as nested PCR-RFLP (0.2%) [24], enrichment double PCR-RFLP-PAGE (0.001%) [25], and RFLP-PAGE-silver staining (0.1%) [26]. The disadvantages of these assays are that multiple steps involved make the whole detection process time-consuming, expensive, and easily contaminated, which does not lend themselves to rapid and cost-effective detection [24]–[26].

At present, microfluidic chips have attracted increasing attention as a powerful platform for biological assays and clinical tests because of their remarkably increased speed, reduced consumption of samples and reagents, and disposability [27], [28]. Considering these distinct advantages, RFLP has been transplanted onto this microscale platform to detect gene mutations [29]–[31]. Some groups have also established on-chip direct endonuclease digestion and electrophoresis detection systems, which demonstrated the capabilities for integration and parallel analysis and the possibility to further increase the detection speed and throughput [32], [33]. However, up to now, quite few reports focused on how to take the unique advantages of microfluidic electrophoresis to achieve highly sensitive screening for low-abundance mutants in cancer biopsies, especially those in trace clinical sample, so detection methods with high sensitivity still remains to be developed to fulfill the clinical and diagnostic utility.

In this study, we established a highly sensitive and efficient RFLP-microfluidic capillary electrophoresis (µCE) platform for the detection of low-abundance KRAS codon 12 mutations by combining the technologies of RFLP and microfluidic chip to subject digested fragments to CE separation. Our self-built sensitive laser-induced fluorescence (LIF) system and the adoption of a new DNA intercalating dye YOYO-1 incrementally enhanced the fluorescence signal. In addition, the utilizing of field amplified sample stacking (FASS) technique during sample introduction further improved the sensitivity. Here, 0.01% of KRAS mutations could be successfully identified with *picoliter*-*scale* sample consumption, and the feasibility for genotyping low-abundance KRAS gene in 98 DNA extracted from paraffin-embedded tissues (PETs) was also explored to indicate its special usefulness in the detection of clinical samples and personalized cancer therapy.

## Materials and Methods

### Ethics Statement

Ninety-eight PETs were obtained from patients with primary CRC at the First Hospital of China Medical University. None of the patients underwent pre-operative radiotherapy or chemotherapy. Ethics review committees in China Medical University approved the study. Written informed consent was provided by all study participants.

### Cell Lines and Clinical Specimens

The human CRC cell lines SW480, HT29, CCL187, LS174T, Clone A, and CX-1 were cultivated at 37°C with 5% CO_2_ in DMEM supplemented with 10% fetal bovine serum. The characteristics of the patients are summarized in Table 1. Median age was 59 years (range, 32–84 years). Male/female ratio was 1.72 (62 males and 36 females). Well, moderately, and low-differentiated adenocarcinomas were present in 35, 57, and 6 patients, respectively. Clinical stage of the patients according to Dukes’ classification was as follows: stage A+B in 56 patients, stage C+D in 42 patients. Lymphatic invasion happened in 26 patients, and distant metastasis occurred in 22 patients. Fifty six patients’ tumors were 5 cm or less, and others’ were more than 5 cm. Ulcer type tumors were found in 67 patients, and eminence type tumors were discovered in the remaining. Forty two tumors were located in colons, and 56 tumors were in rectum.

**Table 1:** K-ras codon 12 mutation rate and clinicopathological parameters.

Category	Subcategory	Total (n)	Direct sequencing (%)	µCE-based RFLP (%)
Age	>50	81	12 (13.3)	27 (33.3) *
	≤50	17	1 (5.9)	1 (5.9)
Gender	Male	62	8 (12.9)	16 (25.8)
	Female	36	5 (13.9)	12 (33.3)
Histology^a^	W. D.	35	6 (17.1)	12 (34.3)
	M. D.	57	7 (12.3)	15 (26.3)
	L. D.	6	0 (0)	1 (16.7)
Dukes stage	A+B	56	5 (8.9)	15 (25)
	C+D	42	8 (19.5)	13 (33.3)
Lymph node metastasis	+	26	6 (23.1)	11 (42.3)
	−	72	7 (9.7)	17 (23.6)
Distant metastasis	+	22	3 (13.6)	3 (13.6)
	−	76	10 (13.2)	25 (32.9)
Tumor size	>5 cm	42	5 (11.9)	11 (26.2)
	≤5 cm	56	8 (14.3)	17 (30.4)
Pathological type	Ulcer type	67	9 (13.4)	18 (26.9)
	Eminence type	31	4 (12.9)	10 (32.3)
Tumor location	Colon	42	8 (19.5)	14 (33.3)
	Rectum	56	5 (8.9)	14 (25)

a W. D.: Well-differentiated adenocarcinoma; M. D.: Moderately differentiated adenocarcinoma; L. D.: Low-differentiated adenocarcinoma.

* P<0.05.

### Genomic DNA Extraction

QIAamp DNA mini kit (*Qiagen, Inc.*) was applied to extract the genomic DNA (gDNA) of cell lines. For the PETs, gDNA was isolated by using the following simple, low-cost protocol. One 10-µm thick section of each block was added to a 1.5 mL micro tube containing 150 µL digestion buffer (50 mM Tris-HCl, pH 8.3; 1 mM EDTA; 1% Tween-20; 500 µg/µL proteinase K). Digestion was performed for 3 h at 56°C with agitation every 30 min. Proteinase K was inactivated by heating for 10 min at 95°C. The supernatant containing gDNA was collected by centrifugation at 13,000 g for 10 min and used as a PCR template.

### RFLP Analysis of KRAS Mutation by Neutral PAGE

**Figure 1:**
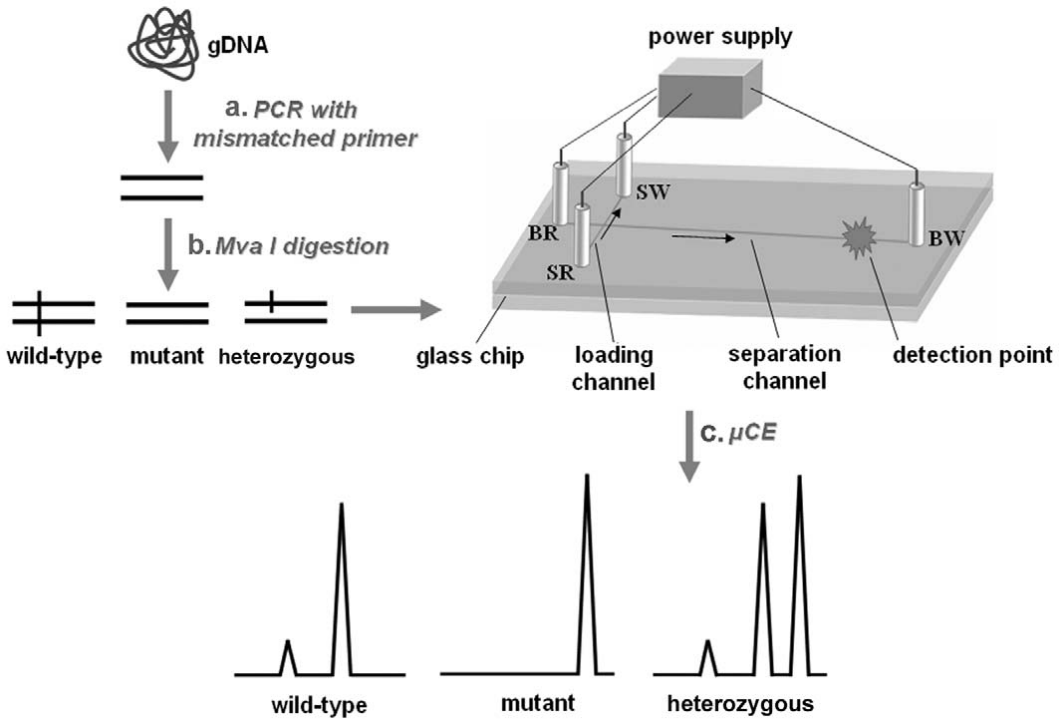
Schematic view of microfluidic capillary electrophoresis-based restriction fragment length polymorphism (µCE-based RFLP) platform. (a) Mismatched primer PCR. A KRAS gene fragment containing codon 12 was amplified from gDNA with mismatched primer, by which a base substitution was introduced to the amplicon and a Mva I restriction endonuclease recognition site was created for wild-type codon 12. (b) Mva I digestion. The amplicon from wild-type template could be cleaved into two fragments, the amplicon from mutant template could not be digested due to the loss of recognition site, and the amplicon from heterozygous template was halfly digested. (c) µCE. The digested amplicon was loaded into microfluidic chip and separated by CE according to the fragment length. The wild type template resolved into two peaks, the mutant template only showed one peak, and the heterozygous template resolved into three peaks. gDNA, genomic DNA. SR: sample reservoir; BR: buffer reservoir; SW: sample waste reservoir; BW: buffer waste reservoir. →, the direction of fluid flow during sample loading and separation modes.

To detect KRAS point mutations in codon 12, a 107-bp fragment of KRAS was amplified from DNA templates extracted from CRC cells or PETs via mismatched primer PCR (Figure 1). The PCR primers used were: forward, 5′-GACTGAATATAAACTTGTGGTAGTTGGACCT-3′, and reverse, 5′-CTATTGTTG GATCATATTCGTCC- 3′. The mismatched forward sense primer was designed to introduce a base substitution that created a Mva I restriction endonuclease (*Fermentas, Inc.*) recognition site for only the wild-type codon 12, so the 107-bp fragment could be completely cleaved into 77- and 30-bp fragments, while amplicon from the mutant template could not be digested due to the loss of recognition site, and the amplicon from heterozygous template was half-digested. The 25 µL PCR reaction mixture contained 0.2 mM dNTPs, 0.5 µM of each primer, 1×PCR buffer (10 mM Tris-HCl, pH 8.3, 1.5 mM MgCl_2_, 50 mM KCl), and 2.5 U Pyrobest DNA polymerase. Total 0.25 µg of gDNA obtained from cells and PETs was used as the templates. PCR was performed by preheating at 94°C for 5 min, followed by 30 cycles of 94°C for 30 s, 65°C for 1 min, 72°C for 1 min, and 72°C for 7 min. The Mva I digestion reaction mixture contained 10 mM Tris-HCl (pH 8.5), 100 mM KCl, 10 mM MgCl_2_, 0.1 mg/mL BSA, 1 unit/µL restriction endonuclease Mva I, and 4 µL of 107 bp PCR product of KRAS. The digestion mixture was incubated at 37°C for 3 h. Then, the digestion product was run in the 5% of neutral PAGE, followed by the staining with ethidium bromide (EtBr).

### Microfluidic Device Fabrication

As shown in Figure 1, a *cross channel glass chip* with a 1.0-cm long sample-loading channel and a 4.5 cm separation channel from the crossing to the buffer waste (BW) reservoir (total length 5.0 cm) was fabricated by wet etching and thermal *bonding* [34]. We used a self-built autofocusing confocal LIF detection device, equipped with a 473 nm diode laser [35]. Fluorescence of the DNA fragments was recorded by a BD11 chart recorder (*Kipp&Zonen, Inc.*). A homemade four-output programmable power supply was used for on-chip sample injection and electrophoretic separation.

### RFLP Analysis of KRAS Mutation by µCE

2-(4-morpholino)ethanesulfonic acid (MES)-Tris buffer at pH 6.1 was prepared by dissolving MES-Tris in water to a concentration of 80 mM MES and 40 mM Tris. The coating solution was made by adding 2% (w/v) of Hydroxypropyl cellulose (HPC, 100,000 MW; *Sigma*-*Aldrich,* St. Louis, MO*)* to the MES-Tris buffer. A 5× Tris-Borate-EDTA (TBE) stock buffer at pH 8.6 was prepared by dissolving Tris-borate-EDTA in water to a concentration of 450 mM Tris, 450 mM borate, and 10 mM EDTA. The sieving solution was made by adding appropriate amount of HPC to different concentration of TBE electrophoresis buffers. Both coating and sieving solutions were stirred thoroughly for 10 min to dissolve the polymer. Following stirring, the solutions were stored overnight at 4°C to remove bubbles, and then filtered twice with a 0.45 µm filter to remove particulates. The DNA intercalating dye YOYO-1 (excitation 473 nm, emission 509 nm) (*Molecular Probes, Inc.*) was added to a concentration of 1 µM in the sieving solution.

All microchannels were rinsed sequentially with water and MES and then filled with coating solution for 12 h. Before using, the coating solution in the channels was expelled and refreshed with the sieving solution using a 5 mL syringe. Ten microliters of sieving solution were added to buffer reservoir (BR), BW, and sample waste reservoir (SW). Different DNA samples including 10 µL of 100-fold diluted ΦX174-HaeIII DNA marker with 11 fragments ranging from 72 to 1353 bp (*TaKaRa, Inc.*) or 10 µL of Mva I digestion product were added into the sample reservoir (SR). Platinum electrodes were inserted into the reservoirs, and the chip was aligned for LIF detection. The sample was loaded using a pinched injection procedure for 80 s. The voltages applied to the BR, SR, SW, and BW were as follows: +200 V, 0 V, +400 V, and +300 V; and during the separation stage, 0 V, +150 V, +150 V, and +600 V/900 V/1200V, were applied. Wild-type templates would resolve into two peaks, mutant templates would show only one peak, and heterozygous templates could produce three peaks (Figure 1). Each sample was run in triples to confirm the results.

### Detection Sensitivity of µCE-based RFLP

To explore the detection sensitivity of this method, gDNA from SW480 cells (which have two mutant alleles at codon 12 of the KRAS gene) was mixed with gDNA from HT29 cells (which have the wild-type KRAS gene) at the decreasing ratios, 1∶1, 1∶10, 1∶10^2^, 1∶10^3^ 1∶10^4^, and 1∶10^5^, respectively. Extracted gDNA was then subjected to PCR, followed by digestion with Mva I and µCE separation.

### Direct Sequencing

KRAS status in codon 12 of the CRC cell lines and PETs were detected by direct sequencing. The following KRAS primers were used: forward, 5′-GTACTGGTGGAGTATTTGATAGTG-3′, and reverse, 5′-AAAGAATGGTCCTGCACCAGTAATA-3′. The PCR was performed with preheating at 95°C for 5 min, followed by 35 cycles at 94°C for 40 seconds, 55°C for 40 seconds, 72°C for 1 min, and a final extension at 72°C for 7 min. All amplicons of the KRAS gene were purified using a DNA purification kit (*Zymo, Inc.*) and directly sequenced using both forward and reverse primers on an ABI 377 sequencer.

### Clone Sequencing

Clone sequencing was performed for the PETs that showed mutation peak profiles in the µCE electropherograms that were not detectable by direct sequencing. The PCR product was purified and inserted into pMD18-T vector and was transformed into E. coli DH5α. The positive recombinant clones were sequenced by using M13 universal primers on an ABI 377 sequencer.

### Statistical Analysis

All statistical analysis employed the Fisher exact test to compare proportions. A two-tailed p value of less than 0.05 was considered statistically significant.

## Results

### Investigation of the Optimal Separation Conditions

**Figure 2:**
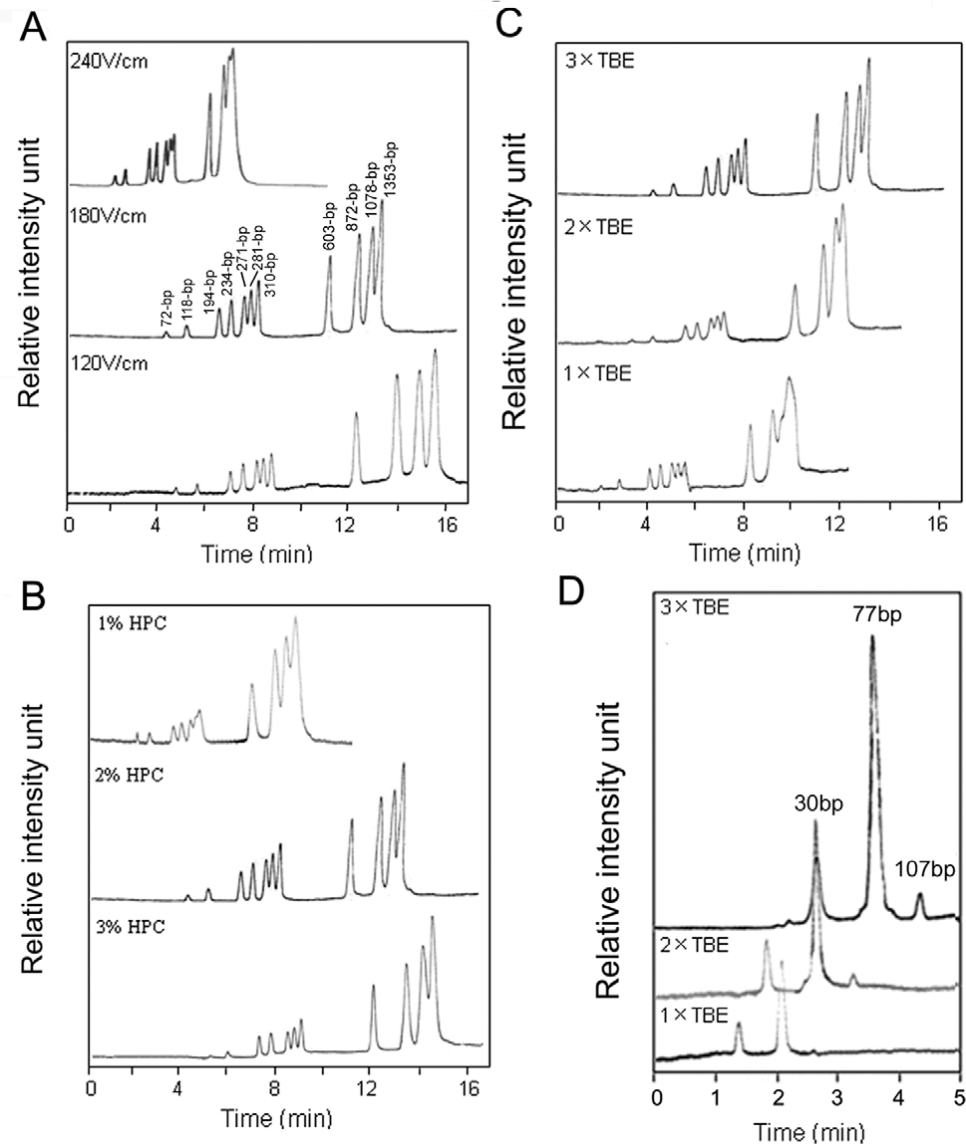
Optimization of separation parameters for the microfluidic capillary electrophoresis-based restriction fragment length polymorphism (µCE-based RFLP) platform. (**A**) The effect of field strength. Conditions for separation were 2% HPC, 3×TBE with varied field strength. (**B**) The effect of polymer concentration. Conditions for separation were 3×TBE, 180 V/cm with varied polymer concentrations. (**C**) The effect of buffer concentration. Conditions for separation were 2% HPC, 180 V/cm with varied ionic strength. (**D**) µCE-based RFLP analysis of the products of enzymatic digestion. Products were separated in sieving buffers with 3×TBE, 2×TBE, or 1×TBE. Separations were performed using 2% HPC under a 180 V/cm electric field.

The electrophoresis conditions were optimized for the best separation efficiency and sensitivity, including separation voltage, sieving matrix (HPC) concentration, and TBE electrophoresis buffer concentration. First, ΦX174 HaeIII DNA Marker containing 11 fragments ranging from 72 to 1353-bp was separated under various electric fields of 240 V/cm, 180 V/cm, and 120 V/cm. The separation under 180 V/cm produced the best combination of resolution and analysis time (Figure 2A). The effect of HPC concentration on the separation is shown in Figure 2B. All the fragments were successfully separated, and the resolution improved as the concentration increased, but when the concentration was higher than 2%, filling the sieving matrix to the micro-channel manually with the syringe became very difficult. Therefore, 2% of HPC was selected as the optimal sieving matrix. Figure 2C demonstrated the effect of the TBE buffer concentration on the separation. As the concentration of TBE increased, the resolution of most DNA fragments were improved, and the peaks of all fragments were slightly higher.

To further optimize the conditions for analyzing digestion products, we separated them without any purification process at 180 V/cm, with 2% HPC in different TBE buffer. All three digestion fragments (30-, 77-, 107-bp) from a mixture in which SW480 cells carrying mutant KRAS gene were in a 1000-fold excess of KRAS wild-type HT29 cells could reach baseline separation. Different from DNA marker, these peaks increased significantly with an increase in TBE buffer concentration from 1× to 3×, and an assay time of 5 min was realized with picoliter-scale sample consumption (Figure 2D).

### Detection Sensitivity and Reproducibility of µCE-RFLP

One-step PCR based RFLP-PAGE, product direct sequencing, and RFLP-µCE were performed individually to compare the sensitivity. The result of neutral PAGE indicated that it could detect 10% of mutant KRAS (Figure 3A), and the PCR product direct sequencing showed a lower detection sensitivity around 20% (Figure 3B). Figure 3C revealed that µCE could identify mutant KRAS in concentrations as low as 0.01% (1∶10^4^). Intra- and inter-assay of µCE detection for the 1∶10^3^ (SW480:HT29) sample demonstrated good reproducibility, and the relative standard deviation (RSD) of migration time reached 2.1% (n  = 3), and 3.1% (n  = 3), respectively (Figure 4), where intra-assay means continual electrophoretic detection without changing the sieving matrix, and inter-assay means that each assay is performed in a newly filled sieving matrix.

**Figure:**
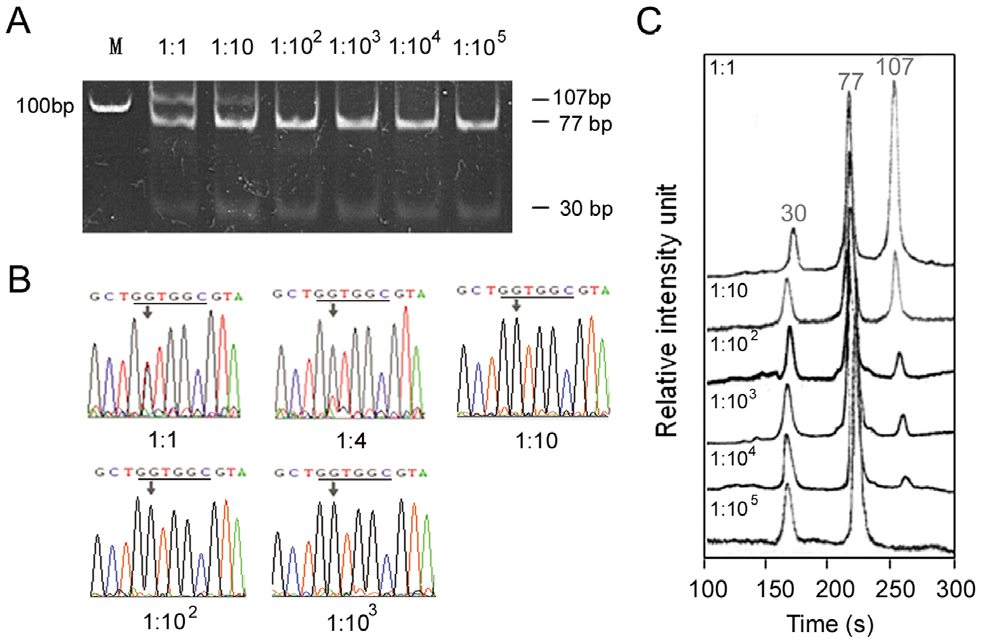
Sensitivity analysis of the µCE-based RFLP platform. SW480 cells carrying mutant KRAS were mixed with KRAS wild-type HT29 cells at various ratios and detected by Natural PAGE (**A**), direct sequencing (**B**), and µCE-based RFLP (**C**), respectively. Conditions for separation were 2% HPC, 3×TBE, and 180 V/cm.

**Figure:**
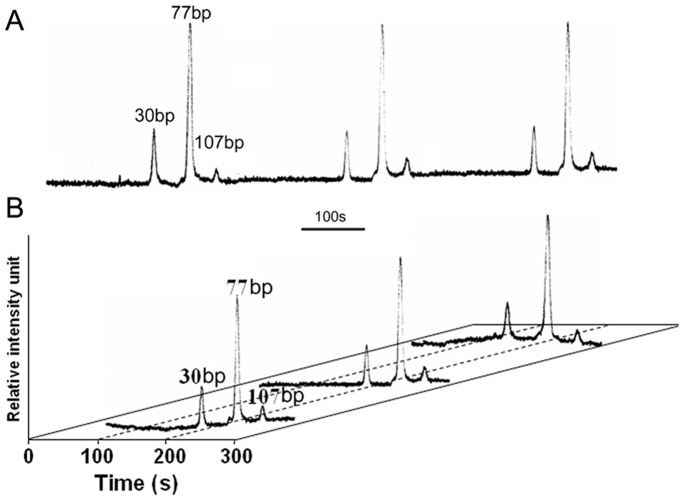
Analysis of reproducibility. (**A**) intra-assay. (**B**) inter-assay.

**Figure 5:**
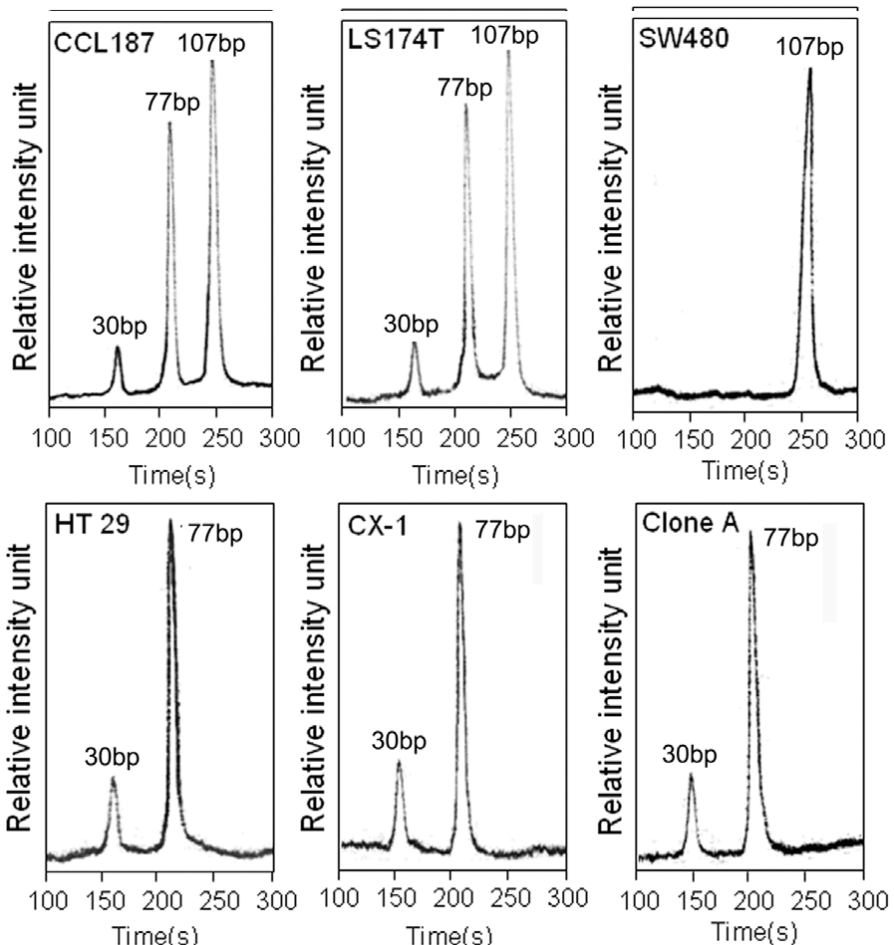
Electropherograms of the KRAS gene in six CRC cell lines.

### Detection of Mutant KRAS in Cell Lines

To study the applicability of this µCE-based RFLP platform, we tested point mutations in codon 12 of KRAS gene from 6 CRC cell lines. The KRAS statuses of these cell lines were all confirmed by direct sequencing. HT29, CX-1 and Clone A cells were revealed to be wild-type; SW480 cells were homozygously mutated; cell lines CCL187 and LS174T were found to be heterozygously mutated (Table 2). Figure 5 showed the electropherograms: HT29, CX-1, and Clone A cells were resolved into two enzymatic-digest fragments of 77- and 30-bp, indicating the absence of mutations; SW480 cells displayed a mutation profile clearly composed of a 107-bp sized major peak; CCL187 and LS174T cells showed two enzymatic-digest fragments (77- and 30-bp) coexisting a 107-bp fragment without restriction site, confirming the coexistence of wild-type and mutant KRAS. The results of using µCE to detect KRAS statuses in 6 CRC cell lines were in complete agreement with those obtained by DNA sequencing.

**Figure 6:**
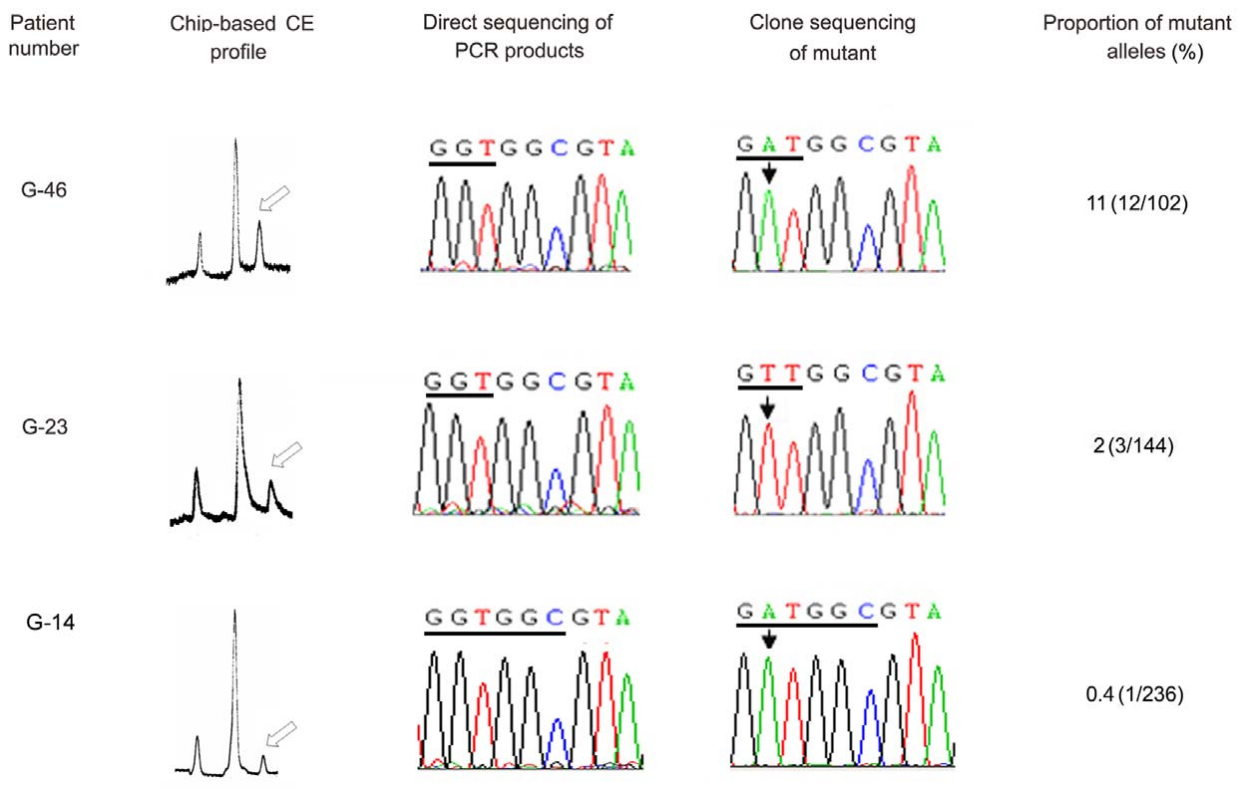
Three representative KRAS gene mutation-positive CRC PETs detected by µCE-based RFLP but not by direct sequencing. The empty arrows indicate the peaks of mutant fragments. The underlined bases in the sequencing results are codons 12 (exon 2) of KRAS. Mutant sites are marked with black arrows.

**Table 2:** Characteristics of the K-ras codon 12 in different colon cancer cells confirmed by sequencing analysis.

Cell name	Mutation status	Mutation position	Mutation type
CCL187	Heterozygous	Codon 12	GGT→GAT
LS174T	Heterozygous	Codon 12	GGT→GAT
SW480	Mutant	Codon 12	GGT→GTT
HT29	Wild-type	–	–
CX-1	Wild-type	–	–
Clone A	Wild-type	–	–

### Detection of Mutant KRAS in Clinical Samples

Here, 98 PETs from CRC patients were analyzed using µCE-based RFLP and PCR product direct sequencing. The results of µCE-based RFLP showed that aberrant electrophoretic migration peaks present in 28 cases, suggesting 28.6% of PETs carried mutations in KRAS codon 12, higher than the frequency obtained by direct sequencing (13, 13.3%). There were 15 KRAS gene mutation-positive PETs detected by µCE-based RFLP, but not by direct sequencing. Clone sequencing confirmed the presence of codon 12 mutation in these samples and verified the accuracy of µCE-based RFLP. The genotypes and proportions of the mutant alleles of all 28 KRAS mutation-positive paraffin-embedded CRC tissues detected by µCE-based RFLP were shown in Table S1. Three representative samples harbored low levels of mutations in KRAS codon 12 were exhibited in Figure 6, and the lowest proportion of mutations was 0.4%, which occurred in PET sample G-14. We compared the clinicopathological data from CRC patients with the mutation status of KRAS in PETs (Table 1). Univariate analysis of the results of µCE-based RFLP revealed that the KRAS mutation in codon 12 was not associated with gender, histology, Dukes stage, lymph node and distant metastasis, tumor size, pathological type, and tumor location, but the patients older than 50 years are much more likely to have KRAS mutations. Direct sequencing didn’t reveal any associations between KRAS mutations and clinical parameters.

## Discussion

KRAS mutation is one of the most *common* oncogenic alterations in various human cancers and plays a very important role in multi-step process of cancer development, including cancer initiation, metastasis, and prognosis [10]. Especially, many recent studies have shown that KRAS codon 12/13 mutations are associated with resistance to the anti-EGFR therapy in mCRC patients and some drug side effects [7]–[10]. Therefore, accurate identification of KRAS status is becoming more essential not only for guiding treatment, but also for reducing side effects and economic burden.

However, it is also very important to note that not all patients currently classified as “wild-type” for KRAS benefit from EGFR-targeted therapies. Although there are many reasons for this, including the involvement of other gene alterations such as loss of PTEN expression and PIK3CA mutation [36], [37], it is most possible that the limited sensitivity of employed detection methods failed to reveal low-abundance mutations, and thus patients were classified incorrectly. Some researchers have reported that methods with high sensitivity could disclose more KRAS mutations, which might increase the possibility of choosing the correct individual therapy [38], [39]. Molinari et al. compared the detection rates of KRAS mutations and the prediction accuracy of anti-EGFR therapy efficacy between direct sequencing and more sensitive method such as mutant-enriched PCR (ME-PCR) and matrix-assisted laser desorption/ionization-time-of-flight (MALDI-TOF) in the same group of mCRC patients, and found these highly sensitive methods detected additional 13 KRAS alterations after direct sequencing, all occurring in nonresponders, which meant the increased sensitivity significantly improved the identification rate of mCRC patients resistant to cetuximab or panitumumab from 39% to 50% [39]. Therefore, more sensitive methods for assessing mutation burden are in a great need, particularly for the assessment of response in biomarker-defined clinical trials.

In this study, we established a novel µCE-based RFLP platform by incorporatingµCE and RFLP to perform sensitive KRAS mutation screening. This platform could reveal 0.01% of CRC cells with mutant KRAS, which was 1 000 times of Sanger sequencing [12], and as least 100 times of most commercially available kits [22], [23], [40]. The detection rate of KRAS mutation in CRC samples was significantly higher than that obtained by direct sequencing; the presence of codon 12 mutation in samples undetectalbe by direct sequencing was all confirmed by clone sequencing, which demonstrated the platform’s capability to detect low-abundance mutations without influencing the accuracy. In addtion, the on-line automatic µCE, staining, recording, and analysis significantly increased detection speed (<5 min) and decreases the risk due to manipulation and cross-contamination in the multiple steps used in conventional gel-based electrophoresis and staining. The assembly of the whole platform was also straightforward and at low cost, which made it readily established in normal molecular labs.

There are some factors attributed to the significantly increased sensitivity in our detection system. At first, a highly sensitive self-built LIF system is applied in combination with a new type of fluorescent dye, YOYO-1. LIF is one of the most sensitive technique available for detection in microfluidic chips, because the coherence and low divergence of a laser beam makes it easy to focus on very small detection volumes and to obtain very high irradiation [41], [42]. YOYO-1 can exhibit up to a 1000-fold increase in fluorescence upon binding to DNA, while the fluorescence of the commonly used EtBr is only enhanced 20–30 fold after intercalation into dsDNA [43], [44]. We also developed a combined acidic buffer-based dynamic and static absorptive coating method to modify the microfluidic channel surface. The results have shown that the coating layer was sufficient to effectively passivate the surface, and therefore gave a high resolution and sensitivity.

Compared with the easy purification of normal PCR products, the digestion fragments from RFLP is usually shorter than 100-bp, dissolved in high salt buffer, and at very low concentration, which make them difficult to purify, recover, and detection. Especially in microfluidic platform, although the picoliter-scale sample assumption due to micro-scaling extremely saved clinical samples and enabled small amount of samples to be analyzed, the main challenging thing is efficient detection of such low analyte concentration. In this work, FASS was employed with some modification to enrich the sample and increase the sensitivity. In FASS, the electric field strength varies inversely with the sample conductivity, so the sample zone with low conductivity has higher field strength than running buffer zone, and the charged analyte velocity will be higher in the sample zone, thus at the buffer interface, analyte ions decelerate abruptly and stack into a narrow and discrete band resulting in sample enrichment [45]–[48]. Feng et al. dissolved a DNA 100-bp marker in three solvents with different ionic strengths: deionized water, 0.2× TBE, and 1× TBE, and separated them in microchips, and found that the DNA dissolved in deionized water gave larger peak area [49], but this won’t work for the RFLP digestion product with high ionic strength. To address this problem, Yang et al. tried to introduce a water plug prior to electrokinetic sample injection and produce a short low-conductivity zone [50], nevertheless this step complicated the CE process and increased the difficulty of operation. Here, we effectively enriched RFLP digestion sample by increasing the difference of ionic strengths between running buffer and samples, and a three-fold increase in peak signal was observed in 3×TBE buffer in the analysis of enzymatic digestion product. This simple modification of FASS eliminated the complicated purification step, facilitated the manipulation of the automatic “lab-on-a-chip”, and enabled the detection of trace quantities of biological samples. In addition, different with conventional CE, the inherently elevated ratio of surface area to the volume of the microfluidic channel facilitated the release of joule heating, so a higher electric field could be employed to enhance the ability for separating the components [51], [52].

Under the optimized condition, KRAS mutations in 98 patients were analyzed. Compared with direct sequencing, the RFLP-µCE achieved a higher detection rate for KRAS codon 12 mutation, 13.3% versus 28.6%. Current reports show that codon 12 mutations are found in 12–30% of Asian patients [53], [54], [55], and in 35–50% of patients from western countries [56], [57], [58]. Our results approached the upper limit of the codon 12 mutation rate in Asian patients, but lower than that of the later. The different mutation rates in different areas and different races might be due to the divergence of environment, dietary habit, and genetic factors.

The results of clinicopathological factors from 98 CRC tissues indicated a strong association between KRAS codon 12 mutation and patient’s age. KRAS mutations in patients older than 50 years (n  = 81) were more frequent than that in the young age groups, which indicated that the older patients would have worse therapeutic effects, and it is necessary for them to take KRAS testing early to update therapy strategy. Other clinicopathological factors, such as gender, histology, Dukes stage, tumor size, pathological type, and location of the tumor showed no positive relationship with KRAS mutations. With the enlargement of sample size, the correlation between KRAS gene and clinicopathological factors may be further disclosed.

In conclusion, the electrophoresis step in RFLP was successfully transferred onto microfluidic chip platform in this study. By using sensitive LIF, new fluorescence dye, optimized electrophoretic conditions, and FASS technique, highly sensitive mutation detection were achieved, with picoliter-scale sample assumption. Especially, the biological samples in high salt concentration such as RFLP digestion products were significantly enriched by FASS, which provided a universal way to detect these types of samples. In the detection of CRC samples, the RFLP-µCE platform remarkably increased the KRAS mutation positive rate, which will be very helpful to accurately identify patients for anti EGFR therapy. The sensitivity of this platform can be further increased by combining with other mutation enrichment techniques in our future work, such as co-amplification at lower denaturation temperature-PCR (COLD-PCR) and peptide nucleic acid (PNA) [59], [60]. Finally, the strategy of RFLP is capable of being employed to detect mutations of any gene sequences as long as there are digestion sites or digestion sites can be generated, thus a large number of different DNA fragments can be analyzed simultaneously by developing continuous sample introduction system or increasing the number of parallel channels on the platform, so that the high-throughput detection can be realized. Considering all of these technological superiorities, the µCE-based RFLP platform posseses a very high potential for more comprehensive application.

## Supporting Information

Table S1 **KRAS mutations in paraffin-embedded colorectal cancer (CRC) tissues.**
(DOC)

## References

[1] Jemal A, Siegel R, Ward E, Hao Y, Xu J, et al (2009) Cancer statistics, 2009. CA Cancer J Clin 59: 225–249. 19474385 10.3322/caac.20006

[2] Van Cutsem E, Köhne CH, Hitre E, Zaluski J, Chang Chien CR, et al (2009) Cetuximab and chemotherapy as initial treatment for metastatic colorectal cancer. N Engl J Med 360: 1408–1417. 19339720 10.1056/NEJMoa0805019

[3] Ma WW, Adjei AA (2009) Novel agents on the horizon for cancer therapy. CA Cancer J Clin 59: 111–137. 19278961 10.3322/caac.20003

[4] Loupakis F, Cremolini C, Salvatore L, Schirripa M, Lonardi S, et al (2012) Clinical impact of anti-epidermal growth factor receptor monoclonal antibodies in first-line treatment of metastatic colorectal cancer: Meta-analytical estimation and implications for therapeutic strategies. Cancer 118: 1523–1532. 22009364 10.1002/cncr.26460

[5] Assenat E, Desseigne F, Thezenas S, Viret F, Mineur L, et al (2011) Cetuximab Plus FOLFIRINOX (ERBIRINOX) as First-Line Treatment for Unresectable Metastatic Colorectal Cancer: A Phase II Trial. Oncologist 16: 1557–1564. 22016477 10.1634/theoncologist.2011-0141 PMC3233290

[6] Mayo SC, Pawlik TM (2009) Current management of colorectal hepatic metastasis. Expert Rev Gastroenterol Hepatol 3: 131–144. 19351284 10.1586/egh.09.8

[7] Karapetis CS, Khambata-Ford S, Jonker DJ, O’Callaghan CJ, Tu D, et al (2008) K-ras mutations and benefit from cetuximab in advanced colorectal cancer. N Engl J Med 359: 1757–1765. 18946061 10.1056/NEJMoa0804385

[8] Amado RG, Wolf M, Peeters M, Van Cutsem E, Siena S, et al (2008) Wild-type KRAS is required for panitumumab efficacy in patients with metastatic colorectal cancer. J Clin Oncol 26: 1626–1634. 18316791 10.1200/JCO.2007.14.7116

[9] Waldner MJ, Neurath MF (2010) The molecular therapy of colorectal cancer. Mol Aspects Med 31: 171–178. 20171980 10.1016/j.mam.2010.02.005

[10] Winder T, Lenz HJ (2010) Molecular predictive and prognostic markers in colon cancer. Cancer Treat Rev 36: 550–556. 20363564 10.1016/j.ctrv.2010.03.005

[11] Fariña Sarasqueta A, Moerland E, de Bruyne H, de Graaf H, Vrancken T, et al (2011) SNaPshot and StripAssay as valuable alternatives to direct sequencing for KRAS mutation detection in colon cancer routine diagnostics. J Mol Diagn 13: 199–205. 21354055 10.1016/j.jmoldx.2010.10.006 PMC3128574

[12] Shackleton M, Quintana E, Fearon ER, Morrison SJ (2009) Heterogeneity in cancer: cancer stem cells versus clonal evolution. Cell 138: 822–829. 19737509 10.1016/j.cell.2009.08.017

[13] Zou H, Taylor WR, Harrington JJ, Hussain FT, Cao X, et al (2009) High detection rates of colorectal neoplasia by stool DNA testing with a novel digital melt curve assay. Gastroenterology 136: 459–470. 19026650 10.1053/j.gastro.2008.10.023

[14] Marchetti A, Gasparini G (2009) K-ras mutations and cetuximab in colorectal cancer. N Engl J Med 360: 833–834. 19228630 10.1056/NEJMc082346

[15] Zhang H, Wang X, Ma Q, Zhou Z, Fang J (2011) Rapid detection of low-abundance K-ras mutation in stools of colorectal cancer patients using chip-based temperature gradient capillary electrophoresis. Lab Invest 91: 788–798. 21242956 10.1038/labinvest.2010.200

[16] Allegra CJ, Jessup JM, Somerfield MR, Hamilton SR, Hammond EH, et al (2009) American Society of Clinical Oncology provisional clinical opinion: testing for KRAS gene mutations in patients with metastatic colorectal carcinoma to predict response to anti-epidermal growth factor receptor monoclonal antibody therapy. J Clin Oncol 27: 2091–1096. 19188670 10.1200/JCO.2009.21.9170

[17] Schimanski CC, Zimmermann T, Schmidtmann I, Gockel I, Lang H, et al (2010) K-ras mutation status correlates with the expression of VEGFR1, VEGFR2, and PDGFRalpha in colorectal cancer. Int J Colorectal Dis 25: 181–186. 19936766 10.1007/s00384-009-0843-7

[18] Scholtka B, Schneider M, Melcher R, Katzenberger T, Friedrich D, et al (2009) A gene marker panel covering the Wnt and the Ras- Raf-MEK-MAPK signalling pathways allows to detect gene mutations in 80% of early (UICC I) colon cancer stages in humans. Cancer Epidemiol 33: 123–129. 19679059 10.1016/j.canep.2009.05.001

[19] Lurje G, Nagashima F, Zhang W, Yang D, Chang HM, et al (2008) Polymorphisms in cyclooxygenase-2 and epidermal growth factor receptor are associated with progression-free survival independent of K-ras in metastatic colorectal cancer patients treated with single-agent cetuximab. Clin Cancer Res14: 7884–7895. 10.1158/1078-0432.CCR-07-5165 19047118

[20] Milano G, Etienne-Grimaldi MC, Dahan L, Francoual M, Spano JP, et al (2008) Epidermal growth factor receptor (EGFR) status and K-Ras mutations in colorectal cancer. Ann Oncol 19: 2033–2038. 18632722 10.1093/annonc/mdn416 PMC2733107

[21] Loda M (1994) Polymerase chain reaction-based methods for the detection of mutations in oncogenes and tumor suppressor genes. Hum Pathol 25: 564–571. 7912220 10.1016/0046-8177(94)90220-8

[22] Cavallini A, Valentini AM, Lippolis C, Campanella D, Guerra V, et al (2010) KRAS genotyping as biomarker in colorectal cancer: a comparison of three commercial kits on histologic material. Anticancer Res 30: 5251–5256. 21187522

[23] Lanthaler AJ, Spizzo G, Mitterer M, Mian C, Mazzoleni G (2011) Interlaboratory comparison of K-ras testing by real-time PCR and RFLP in colorectal cancer samples. Diagn Mol Pathol 20: 90–93. 21532493 10.1097/PDM.0b013e31820e5f9a

[24] Xiao SX, Wu L, Wang DB, Hu JG, Liu HP, et al (1997) Detection of mutated K-ras at codon 12 with a modified PCR-RFLP technique. Prog Biochem Biophys 24: 375–378.

[25] Schimanski CC, Linnemann U, Berger MR (1999) Sensitive detection of K-ras mutations augments diagnosis of colorectal cancer metastases in the liver. Cancer Res 59: 5169–5175. 10537293

[26] Nishikawa T, Maemura K, Hirata I, Matsuse R, Morikawa H, et al (2002) A simple method of detecting K-ras point mutations in stool samples for colorectal cancer screening using one-step polymerase chain reaction/restriction fragment length polymorphism analysis. Clin Chim Acta 318: 107–112. 11880119 10.1016/s0009-8981(01)00806-3

[27] Kleparník K, Bocek P (2007) DNA diagnostics by capillary electrophoresis. Chem Rev 107: 5279–5317. 17973434 10.1021/cr0101860

[28] Ohno K, Tachikawa K, Manz A (2008) Microfluidics: applications for analytical purposes in chemistry and biochemistry. Electrophoresis 29: 4443–4453. 19035399 10.1002/elps.200800121

[29] Akamine R, Yatsushiro S, Yamamura S, Kido J, Shinohara Y, et al (2009) Direct endonuclease digestion and multi-analysis of restriction fragment length polymorphisms by microchip electrophoresis. J Pharm Biomed Anal 50: 947–953. 19616912 10.1016/j.jpba.2009.06.034

[30] Minucci A, Delibato E, Castagnola M, Concolino P, Ameglio F, et al (2008) Identification of RFLP G6PD mutations by using microcapillary electrophoretic chips (Experion). J Sep Sci 31: 2694–2700. 18693312 10.1002/jssc.200800216

[31] Qin J, Liu Z, Wu D, Zhu N, Zhou X, et al (2005) Genotyping the -6A/G functional polymorphism in the core promoter region of angiotensinogen gene by microchip electrophoresis. Electrophoresis 26: 219–224. 15624175 10.1002/elps.200406158

[32] Chowdhury J, Kagiala GV, Pushpakom S, Lauzon J, Makin A, et al (2007) Microfluidic platform for single nucleotide polymorphism genotyping of the thiopurine S-methyltransferase gene to evaluate risk for adverse drug events, J Mol Diagn. 9: 521–529. 10.2353/jmoldx.2007.070014 PMC1975104 17690215

[33] Nachamkin I, Panaro NJ, Li M, Ung H, Yuen PK, et al (2001) Agilent 2100 bioanalyzer for restriction fragment length polymorphism analysis of the Campylobacter jejuni flagellin gene. J Clin Microbiol 39: 754–757. 11158144 10.1128/JCM.39.2.754-757.2001 PMC87813

[34] Jia ZJ, Fang Q, Fang ZL (2004) Bonding of glass microfluidic chips at room temperatures. Anal Chem 76: 5597–5602. 15362926 10.1021/ac0494477

[35] Fang Q, Xu GM, Fang ZL (2002) A high-throughput continuous sample introduction interface for microfluidic chip-based capillary electrophoresis systems. Anal Chem 74: 1223–1231. 11922288 10.1021/ac010925c

[36] Sood A, McClain D, Maitra R, Basu-Mallick A, Seetharam R, et al (2012) PTEN gene expression and mutations in the PIK3CA gene as predictors of clinical benefit to anti-epidermal growth factor receptor antibody therapy in patients with KRAS wild-type metastatic colorectal cancer. Clin Colorectal Cancer 11: 143–150. 22285706 10.1016/j.clcc.2011.12.001 PMC3350566

[37] Mao C, Zhou J, Yang Z, Huang Y, Wu X, et al (2012) KRAS, BRAF and PIK3CA mutations and the loss of PTEN expression in Chinese patients with colorectal cancer. PLoS One 7: e36653. 22586484 10.1371/journal.pone.0036653 PMC3346734

[38] Weidlich S, Walsh K, Crowther D, Burczynski ME, Feuerstein G, et al (2011) Pyrosequencing-based methods reveal marked inter-individual differences in oncogene mutation burden in human colorectal tumours. Br J Cancer 105: 246–254. 21712828 10.1038/bjc.2011.197 PMC3142798

[39] Molinari F, Felicioni L, Buscarino M, De Dosso S, Buttitta F, et al (2011) Increased detection sensitivity for KRAS mutations enhances the prediction of anti-EGFR monoclonal antibody resistance in metastatic colorectal cancer. Clin Cancer Res 17: 4901–4914. 21632860 10.1158/1078-0432.CCR-10-3137

[40] Angulo B, García-García E, Martínez R, Suárez-Gauthier A, Conde E, et al (2010) A commercial real-time PCR kit provides greater sensitivity than direct sequencing to detect KRAS mutations: a morphology-based approach in colorectal carcinoma. J Mol Diagn 12: 292–299. 20203003 10.2353/jmoldx.2010.090139 PMC2860464

[41] Götz S, Karst U (2007) Recent developments in optical detection methods for microchip separations. Anal Bioanal Chem 387: 183–192. 17031620 10.1007/s00216-006-0820-8 PMC7080113

[42] Jiang GF, Attiya S, Ocvirk G, Lee WE, Harrison DJ (2000) Red diode laser induced fluorescence detection with a confocal microscope on a microchip for capillary electrophoresis. Biosens Bioelectron 14: 861–869. 10945461 10.1016/s0956-5663(99)00056-1

[43] Benson SC, Mathies RA, Glazer AN (1993) Heterodimeric DNA-binding dyes designed for energy transfer: stability and applications of the DNA complexes. Nucleic Acids Res 21: 5720–5726. 8284220 10.1093/nar/21.24.5720 PMC310540

[44] Benson SC, Mathies RA, Glazer AN (1993) Heterodimeric DNA-binding dyes designed for energy transfer: stability and applications of the DNA complexes. Nucleic Acids Res 21: 5727–5735. 8284220 10.1093/nar/21.24.5720 PMC310540

[45] Gebauer P, Bocek P (2009) Electrophoretic sample stacking. Electrophoresis30: S27–S33. 10.1002/elps.200900053 19517514

[46] Beard NP, Zhang CX, deMello AJ (2003) In-column field-amplified sample stacking of biogenic amines on microfabricated electrophoresis devices. Electrophoresis 24: 732–739. 12601745 10.1002/elps.200390088

[47] Malá Z, Krivánková L, Gebauer P, Bocek P (2007) Contemporary sample stacking in CE: a sophisticated tool based on simple principles. Electrophoresis 28: 243–253. 17136738 10.1002/elps.200600397

[48] Malá Z, Slampová A, Gebauer P, Bocek P (2009) Contemporary sample stacking in CE. Electrophoresis 30: 215–229.J Chromatogr A. 1051: 147–153. 10.1002/elps.200800433 19101931

[49] Xu F, Jabasini M, Zhu B, Ying L, Cui X, et al (2004) Single-step quantitation of DNA in microchip electrophoresis with linear imaging UV detection and fluorescence detection through comigration with a digest. J Chromatogr A 1051: 147–153. 15532567 10.1016/j.chroma.2004.05.019

[50] Yang Y, Boysen RI, Hearn MT (2006) Optimization of field-amplified sample injection for analysis of peptides by capillary electrophoresis-mass spectrometry. Anal Chem 78: 4752–4758. 16841892 10.1021/ac051735v

[51] Petersen NJ, Nikolajsen RP, Mogensen KB, Kutter JP (2004) Effect of Joule heating on efficiency and performance for microchip-based and capillary-based electrophoretic separation systems: a closer look. Electrophoresis 25: 253–269. 14743478 10.1002/elps.200305747

[52] Evenhuis CJ, Haddad PR (2009) Joule heating effects and the experimental determination of temperature during CE. Electrophoresis 30: 897–909. 19197907 10.1002/elps.200800643

[53] Bornholdt J, Hansen J, Steiniche T, Dictor M, Antonsen A, et al (2008) K-ras mutations in sinonasal cancers in relation to wood dust exposure. BMC Cancer 8: 53. 18289366 10.1186/1471-2407-8-53 PMC2278146

[54] Ko JM, Cheung MH, Wong CM, Lau KW, Tang CM, et al (1998) Ki-ras codon 12 point mutational activation in Hong Kong colorectal carcinoma patients.Cancer Lett. 134: 169–176. 10.1016/s0304-3835(98)00257-2 10025877

[55] Wu CM, Tang R, Wang JY, Changchien CR, Hsieh LL (2005) Frequency and spectrum of K-RAS codons 12 and 13 mutations in colorectal adenocarcinomas from Taiwan. Cancer Genet Cytogenet 158: 55–60. 15771905 10.1016/j.cancergencyto.2004.08.030

[56] Capella G, Cronauer-Mitra S, Pienado MA, Perucho M (1991) Frequency and spectrum of mutations at codons 12 and 13 of the c-K-ras gene in human tumors. Environ Health Perspect 93: 125–131. 1685441 10.1289/ehp.9193125 PMC1568052

[57] Delattre O, Olschwang S, Law DJ, Melot T, Remvikos Y, et al (1989) Multiple genetic alterations in distal and proximal colorectal cancer. Lancet 2: 353–356. 2569552 10.1016/s0140-6736(89)90537-0

[58] Oudejans JJ, Slebos RJ, Zoetmulder FA, Mooi WJ, Rodenhuis S (1991) Differential activation of ras genes by point mutation in human colon cancer with metastases to either lung or liver. Int J Cancer 49: 875–879. 1959991 10.1002/ijc.2910490613

[59] Li J, Wang L, Mamon H, Kulke MH, Berbeco R, et al (2008) Replacing PCR with COLD-PCR enriches variant DNA sequences and redefines the sensitivity of genetic testing. Nat Med 14: 579–584. 18408729 10.1038/nm1708

[60] Luo JD, Chan EC, Shih CL, Chen TL, Liang Y, et al (2006) Detection of rare mutant K-ras DNA in a single-tube reaction using peptide nucleic acid as both PCR clamp and sensor probe. Nucleic Acids Res 34: e12. 16432256 10.1093/nar/gnj008 PMC1345699

