# Supplementary material for: Detection of Low-Abundance KRAS Mutations in Colorectal Cancer Using Microfluidic Capillary Electrophoresis-Based Restriction Fragment Length Polymorphism Method with Optimized Assay Conditions
Source: PLoS One. 2013 Jan 23;8(1):e54510. doi: 10.1371/journal.pone.0054510 (PMC3552804; doi:10.1371/journal.pone.0054510)
Supplement: Table S1 — KRAS mutations in paraffin-embedded colorectal cancer (CRC) tissues. (DOC) [file pone.0054510.s001.doc]

Table S1 KRAS mutations in paraffin-embedded colorectal cancer (CRC) tissues.

| Patient | μCE-based RFLP | Direct sequencing | Clone sequencing | Proportion of mutant alleles (%) |
| --- | --- | --- | --- | --- |
| 1 | + | 2G→A^a^ | — | 29 |
| 2 | + | 2G→A | — | 22 |
| 3 | + | 2G→A | — | 31 |
| 4 | + | 1G→T | — | 42 |
| 5 | + | 1G→A | — | 22 |
| 6 | + | 1G→A | — | 24 |
| 7 | + | 2G→A | — | 36 |
| 8 | + | 2G→A | — | 33 |
| 9 | + | 2G→A | — | 34 |
| 10 | + | 2G→C | — | 20 |
| 11 | + | 2G→A | — | 19 |
| 12 | + | 2G→A | — | 17 |
| 13 | + | 2G→A | — | 46 |
| 14 | + | WT | 2G→A | 11 |
| 15 | + | WT | 2G→A | 7 |
| 16 | + | WT | 2G→T | 14 |
| 17 | + | WT | 1G→T | 2 |
| 18 | + | WT | 2G→A | 5 |
| 19 | + | WT | 2G→A | 9 |
| 20 | + | WT | 2G→T | 8 |
| 21 | + | WT | 2G→T, 3C→T^b^ | 17 |
| 22 | + | WT | 2G→A | 11 |
| 23 | + | WT | 2G→T | 10 |
| 24 | + | WT | 2G→A | 0.4 |
| 25 | + | WT | 2G→A, 2G→T | 3 |
| 26 | + | WT | 2G→T | 2 |
| 27 | + | WT | 2G→A | 12 |
| 28 | + | WT | 2G→A | 11 |

^a^1G and 2G indicate the first and second bases on codon 12.

^a^3C indicate the third bases on codon 15.

WT, wild-type. —, not analyzed. +, mutant KRAS detected by μCE-based RFLP.
